# Analysis of the Characteristics of TIGIT-Expressing CD3^−^CD56^+^NK Cells in Controlling Different Stages of HIV-1 Infection

**DOI:** 10.3389/fimmu.2021.602492

**Published:** 2021-02-26

**Authors:** Xin Zhang, Xiaofan Lu, Allen Ka Loon Cheung, Qiuyue Zhang, Zhiying Liu, Zhen Li, Lin Yuan, Rui Wang, Yan Liu, Bin Tang, Huan Xia, Hao Wu, Tong Zhang, Bin Su

**Affiliations:** ^1^Department of Infectious Diseases and Medical Immunology, Beijing Youan Hospital, Capital Medical University, Beijing, China; ^2^Beijing Key Laboratory for HIV/AIDS Research, Beijing, China; ^3^Department of Biology, Faculty of Science, Hong Kong Baptist University, Hong Kong, China

**Keywords:** HIV-1, immune responses, immune exhaustion, TIGIT, NK cells

## Abstract

TIGIT expression on natural killer (NK) cells is associated with dysfunction during chronic HIV infection, but the phenotype and biological functions of these cells in the context of acute HIV-1 infection remain poorly understood. Here, 19 acutely infected HIV-1 patients traced at first, third and twelfth month, and age-matched patients with chronic HIV-1 infection were enrolled to investigate the phenotype and functions of TIGIT expression on NK cells. We found that TIGIT-expressing NK cells did not increase in frequency in the first, third and twelfth month of infection until chronic HIV-1 infection lasted over 2 years. The number of TIGIT^+^NK cells in acute infection was positively associated with HIV-1 viral load (*r* = 0.53, *P* = 0.0009). CD96 was significantly upregulated on NK cells after acute infection for 1 month and in chronic infection over 2 years, while CD226 was downregulated in chronic infection over 2 years. Further, at different stages of infection, CD96^−^CD226^+^ cells diminished among total NK cells, TIGIT^+^NK and TIGIT^−^NK cells, while CD96^+^CD226^−^ cells expanded. Reduced CD96^−^CD226^+^ cells and elevated CD96^+^CD226^−^ cells among NK cells especially TIGIT^−^NK cells, had opposite associations with viral load in the first month of infection, as well as CD4 T-cell counts in including the twelfth month and more than 2 years of chronic infection. In both HIV-1-infected individuals and healthy donors, TIGIT was predominantly expressed in NKG2A^−^NKG2C^+^NK cells, with a significantly higher proportion than in NKG2A^+^NKG2C^−^NK cells. Moreover, the frequencies of TIGIT^+^NK cells were positively associated with the frequencies of NKG2A^−^NKG2C^+^NK cells in acute infection (*r* = 0.62, *P* < 0.0001), chronic infection (*r* = 0.37, *P* = 0.023) and healthy donors (*r* = 0.36, *P* = 0.020). Enhanced early activation and coexpression of CD38 and HLA-DR in TIGIT^+^NK cells were detected compared to TIGIT^−^NK cells, both of which were inversely associated with the decrease in CD4 T-cell counts in both acute and chronic HIV-1 infection. The ability of TIGIT^+^NK cells to produce TNF-α, IFN-γ and CD107a degranulation substance were consistently weaker than that of TIGIT^−^NK cells in both acute and chronic infection. Moreover, the functionalities of TIGIT^+^NK cells were lower than those of TIGIT^−^NK cells, except for TNF-α^−^CD107a^+^IFN-γ^−^NK cells. These findings highlight the phenotype and functional characteristics of TIGIT-expressing NK cells which have poor capabilities in inhibiting HIV-1 replication and maintaining CD4 T-cell counts.

## Introduction

Human natural killer (NK) cells are important innate immune cells and play a key role in host defense due to their capacity to mediate cytotoxicity and to release cytokines for lysing tumor cells and virus-infected cells. They can be subdivided into three subsets, CD3^−^CD16^+^CD56^+^, CD3^−^CD16^−^CD56^+^, and CD3^−^CD16^+^CD56^−^. CD3^−^CD16^+^CD56^+^NK cells predominate among total NK cells (≥90%) compared to the CD56^−^CD16^+^NK cells in low abundance (1). In human immunodeficiency virus type-1 (HIV-1) infection, peripheral blood CD3^−^CD56^dim^CD16^+^NK cells decreases while the frequency of CD3^−^CD56^bright^CD16^+^NK cells increases with disease progression (2).

Preceded by the establishment of HIV-1-specific adaptive immune responses, NK cells, as crucial innate immune effector cells, become rapidly activated and expanded in response to the initial phase of HIV-1 replication (3). NK cell activation is regulated by the balance between activating and inhibitory receptors and by the cytokine milieu in the environment (4). IL-12, IL-15, and IL-18, which are secreted by activated dendritic cells and macrophages in response to HIV-1 (5–7), may synergistically activate NK cells in the acute phase of infection.

T-cell immunoreceptor with Ig and ITIM domain (TIGIT) was recently identified as an inhibitory receptor expressed mainly on CD8^+^ T cells (8), CD4^+^ T cells (9) and NK cells but not on dendritic cells, macrophages, or B lymphocytes (10). TIGIT is an immune checkpoint receptor thought to be involved in mediating T cell and NK cell exhaustion in tumors through its interaction with the poliovirus receptor (PVR, CD155) (11–14). Blocking of the TIGIT/PVR interaction prevents NK cell exhaustion and elicits potent antitumor immunity by enhancing the capacity of NK cells to release cytokines and degranulation substances (13, 15, 16). Additionally, TIGIT expression on NK cells displays wide variation in peripheral blood mononuclear cells (PBMCs) among healthy individuals (approximately 30~90%), and it has been related to the phenotypic and functional heterogeneity of NK cells (17). The levels of TIGIT expression on NK cells show wide variation, and NK cells with low TIGIT expression perform relatively high levels of cell functions compared to NK cells with high TIGIT expression (17). Whereas, TIGIT blockade did not seem to improve NK-cell responses to HIV-infected CD4 T cells in healthy donors (18).

In the context of the anti-HIV immune response, upregulated TIGIT expression on NK cells correlates inversely with CD4 T-cell counts and positively with plasma viral loads by reducing the NK production of interferon-gamma (IFN-γ) (19). CD226, an activating receptor, competing the common ligand of CD155 with TIGIT and CD96, counterbalances the inhibitory CD96 and TIGIT receptors which are novel immune checkpoint targets for cancer immunotherapy (20–22). CD96^+^NK cells are more severely dysfunctional compared with CD96^−^NK cells (23, 24), as evidenced by lowered expression of IFN-γ and TNF-α, and increased gene expression levels of IL-10 and TGF-β. CD226 endows educated NK cells with enhanced effector functions but is dispensable for education. TIGIT and CD226 usually expressed on opposing NK cell subsets, where CD226 is higher on licensed NK cells, and TIGIT on unlicensed NK cells (25). Furthermore, the expression of TIGIT on CD226^+^NK cells was significantly higher than on CD226^−^NK cells in HIV-infected individuals (19). Though many studies have investigated TIGIT expression and function in T lymphocytes, current understanding of TIGIT expression and function in NK cells, especially in acute HIV-1 infection (AHI) is lacking.

In this study, we demonstrated the dynamics of TIGIT, CD96 and CD226 expression and functions in NK cells in the first, third and twelfth month after HIV-1 infection in our Beijing PRIMO clinical cohort and chronic HIV-1 individuals with over 2 years of infection. TIGIT expression on NK cells did not frequently increase in the early phase of infection, though the frequency of TIGIT-expressing NK cells was positively associated with HIV-1 viral load. The functionalities of TIGIT^+^NK cells were found to be decreased compared to those of TIGIT^−^NK cells, which indicates that TIGIT expression on NK cells impaired the ability of NK in inhibiting HIV-1 replication.

## Materials and Methods

### Subjects

The subjects in this study were recruited from the Beijing PRIMO clinical cohort, a prospective study cohort of HIV-negative men who have sex with men (MSM) designed to identify cases of acute HIV-1 infection at Beijing Youan Hospital, Beijing, China; the cohort was established in October 2006. The enrolled subjects were monitored every 2 months for anti-HIV antibodies, HIV RNA levels, and clinical signs of acute/early infection, as previously described (26). From the detection of seroconversion, peripheral whole blood collection was conducted at weeks 1, 2, 4, 8, and 12 and then every 3 months thereafter, and PBMCs were isolated and cryopreserved. Subjects with an anti-HIV antibody negative or indeterminate result but positivity by the nucleic acid amplification test were defined as acutely infected with HIV (27). Alternatively, acute HIV infection was estimated to have occurred in the midpoint between the last seronegative and the first seropositive result. The date of acquisition of HIV infection was defined as follows: (1) HIV infection occurring 14 days before the first sample was found to be positive for HIV RNA but negative for the anti-HIV antibody (27); (2) subjects with an indeterminate western blot result were estimated to have been infected 30 days prior to the index or enrollment specimen; and (3) subjects who were negative for the anti-HIV antibody and negative for HIV-1 RNA followed by seropositivity and RNA positivity with a time between tests <2 months (27–29).

Nineteen individuals from the Beijing PRIMO cohort who did not receive antiretroviral treatment during the 1^st^ year of HIV-1 infection were enrolled in this study (identified around 2009); of these, 17 underwent three visit time points at months 1, 3, and 12, and 2 underwent two visit time points at months 3 and 12. Meanwhile, chronic individuals who were infected with HIV-1 for at least 2 years and did not receive antiretroviral treatment were also enrolled in this study. Age-matched HIV-1-uninfected individuals were enrolled as controls.

### Ethics Statement

All relevant experiments in this study were approved by the Beijing Youan Hospital Research Ethics Committee, and written informed consent was obtained from each participant in accordance with the Declaration of Helsinki. All participants provided written informed consent for the collection of information, and their clinical samples were stored and used for research. The methods used conformed to approved guidelines and regulations.

### Flow Cytometry Staining of Surface and Intracellular Markers

Thawed PBMCs in RPMI 1640 medium were stained with antibodies against CD3-Percp (HIT3a, Biolegend), CD56-BV786 (NCAM, BD Bioscience), CD69-APC/Fire™ 750 (FN50, Biolegend), TIGIT-PE/Cyanine7 (cloneA15153G, Biolegend), CD159a-FITC (REA110, Miltenyi Biotec), CD159c-PE (REA205, Miltenyi Biotec), CD38-Alexa Fluor® 700 (HIT2, Biolegend), and HLA-DR- Brilliant Violet 650™ (L243, Biolegend). NK cells were also stained with the following antibodies including CD3-APC/Cyanine7 (OKT3, Biolegend), CD16- Percp Cyanine5.5 (3G8, Biolegend), CD56-FITC (HCD56, Biolegend), CD96-PE (NK92.39, Biolegend), CD226-APC (11A8, Biolegend), TIGIT-Brilliant Violet 421™ (A15153G, Biolegend); CD155-PE (SKII.4, Biolegend), CD4 (PE/Cyanine7, Biolegend). Another panel of NK cell-specific antibodies, including anti-CD3-APC/Cyanine7 (OKT3, Biolegend), -CD56-BV786 (NCAM, BD Bioscience), and -TIGIT-PE/Dazzle™ 594 (A15153G, Biolegend), were stained for 20 minutes at room temperature. After the PBMCs were washed, fixed and permeabilized with Foxp3/Transcription Factor staining buffer set (Cat: 00-5523-00, eBioscience™) for 40 minutes at 4°C, Ki-67-Brilliant Violet 711™ (Ki-67, Biolegend) were added for staining for 30 min at 4°C. Isotype control mAbs were purchased from the corresponding companies. Cytometer setup and tracking (CST) calibration particles were used to ensure that fluorescence intensity measurements were consistent in all experiments. After the PBMCs were washed with PBS buffer and fixed with 4% formaldehyde solution, the cells were assessed using a BD Fortesa flow cytometer, and dead cells were excluded by staining with LIVE/DEAD fixable viability stain 510. The data were analyzed with FlowJo Software version 10.0 (Treestar, Ashland, OR, USA).

### Intracellular Cytokines and Degranulation Substance Staining for NK Cells

PBMCs were thawed and incubated overnight in RPMI 1,640 medium (HyClone, Logan, UT, USA) supplemented with 10% fetal bovine serum (HyClone), 50 IU/ml penicillin-streptomycin (HyClone), and 2 mM L-glutamine (HyClone) and cultured with 500 U/ml IL-2 (R&D), 20 ng/ml IL-12 (R&D) and 50 ng/ml IL-15 (R&D) for approximately 24 h (h). Then, the cultured PBMCs were used to kill K562 target cells at a ratio of 10:1 or without target K562 cells as a negative control. An anti-CD107a-FITC (H4A3, Biolegend) antibody was incubated with these cells at the same time. After 1 h, 3 μg/ml brefeldin A and 2 μM monensinagents (eBioscience™ 1000 × ) were added to the cells and incubated for 5 h at 37°C. The PBMCs were stained with fluorescence-conjugated human monoclonal antibodies (mAbs), including CD3-Percp-Cy5.5 (HIT3a, Biolegend), CD56-APC/Cyanine7 (HCD56, Biolegend), and TIGIT-Brilliant Violet 421™ (A15153G, Biolegend), for 20 min at room temperature, fixed and permeabilized with BD FACS™ permeabilizing solution (Cat: 340457). Intracellular staining was performed for interferon gamma (IFN-γ)-PE and TNF-α-PE-Cy7 for 30 min at 4°C. Isotype control mAbs were purchased from the corresponding companies. The stained cells were evaluated using a BD Fortesa flow cytometer, and dead cells were excluded by staining with LIVE/DEAD fixable viability stain 510; the data were analyzed with FlowJo Software version 10.0 (Treestar, Ashland, OR, USA).

### CD4 T-Cell Count and Viral Load Measurement

Routine blood CD4 T-cell counts (cells/μl) were measured by four-color flow cytometry with human CD45, CD3, CD4, and CD8 cell markers (BD Biosciences) in FACS lysing solution (BD Biosciences) according to the manufacturer's instructions using peripheral whole-blood samples from each patient. The plasma HIV-1 viral load (copies/ml of plasma) was quantified by real-time PCR (Abbott Molecular Inc., Des Plaines, IL, USA). This assay has a sensitivity of 40 copies/ml of plasma for viral RNA detection. The viral load set point at the very early stage of HIV-1 infection was calculated and reported in a previous study (26).

### Statistical Analysis

Statistical analysis was performed with GraphPad Prism software version 5.03 (GraphPad Software, San Diego, California, USA). Differences were analyzed using ANOVA or the Kruskal-Wallis test for multiple groups comparison, Student's *t*-tests (unpaired *t*-test for unpaired variables and paired *t*-test for paired variables) or non-parametric Mann–Whitney *U* tests for two nonparametric variables. Wilcoxon signed rank test was used to analyze paired variables. Spearman's rank correlation analysis was performed to assess the relationship between two variables. Differences were considered statistically significant at *P* < 0.05 in two-tailed tests. The detailed statistical analysis is described in the figure legends.

## Results

### TIGIT^+^NK Cells Did Not Increase During Acute HIV-1 Infection

**Figure 1:**
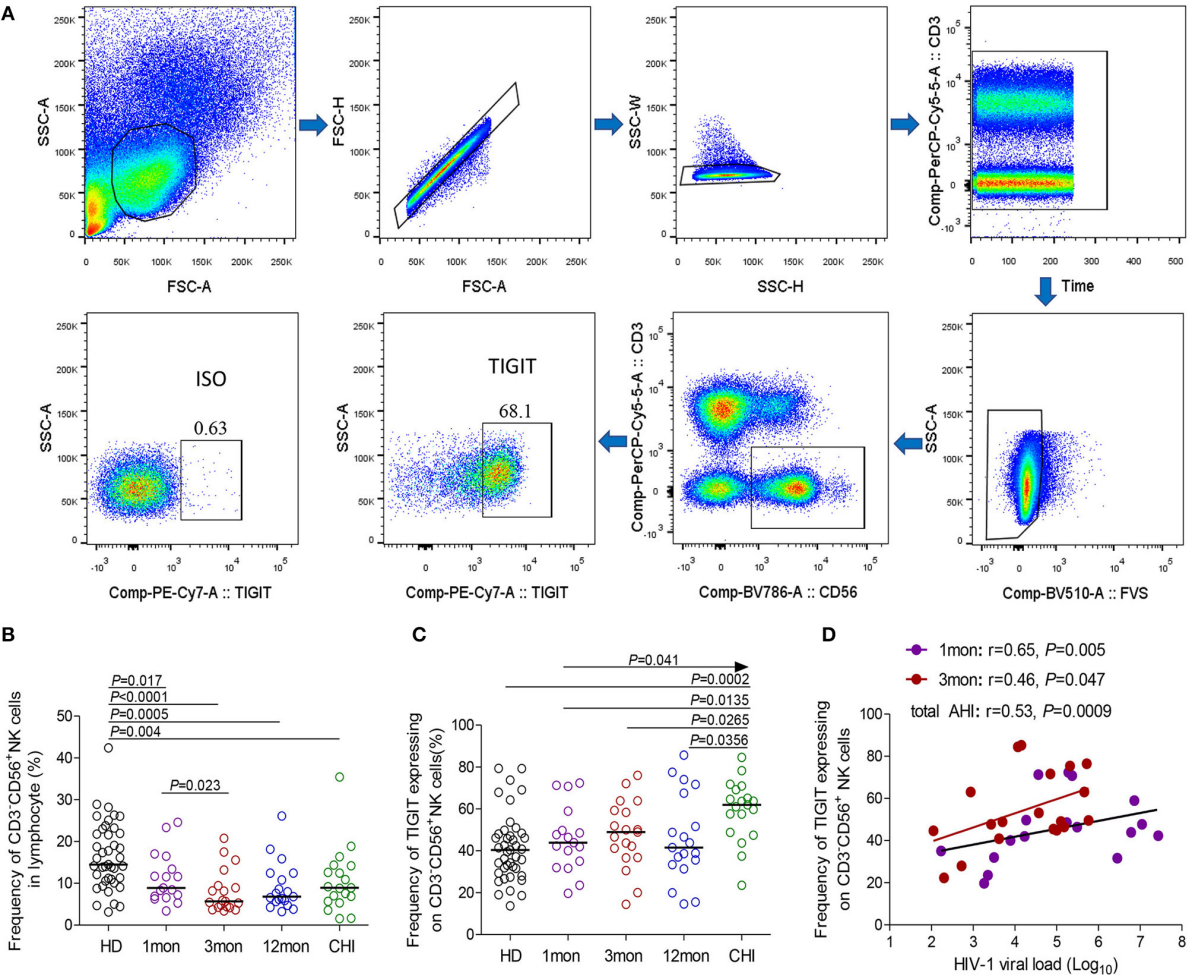
TIGIT expression on NK cells at different stages of HIV-1 infection. **(A)** Flow cytometer charts of TIGIT expression on CD3^−^CD56^+^NK cells; **(B)** Change of the frequency of CD3^−^CD56^+^NK cells; **(C)** Change of the proportion of TIGIT^+^CD3^−^CD56^+^NK cells; **(D)** Correlation of TIGIT expressing on NK cells with HIV-1 viral load; Arrow means the multiple groups comparison by the Kruskal–Wallis test; HD: healthy donors; 1, 3, 12mon, CHI: the first, third, twelfth month of HIV-1 infection, and chronic HIV-1 infection over 2 years, respectively.

Flow cytometry analysis of NK cells was performed as shown in Figure 1A. Based on the data in Figure 1B, the proportion of CD3^−^CD56^+^NK cells in lymphocytes decreased in the first (*P* = 0.017), third (*P* < 0.0001) and twelfth month (*P* = 0.0005) after the onset of HIV-1 infection and also in chronic HIV-1 infection over 2 years (*P* = 0.004). Compared with healthy individuals, TIGIT expression on CD3^−^CD56^+^NK cells significantly increased in chronic HIV-1 infection over 2 years (*P* = 0.0002) but not in the first, third, or twelfth month after the onset of HIV-1 infection (Figure 1C). The amounts of TIGIT^+^NK cells were positively associated with the HIV-1 viral load in the first and third months after HIV-1 infection, as shown in Figure 1D (first month: *r* = 0.65, *P* = 0.005; third month: *r* = 0.46, *P* = 0.047). These results indicated that TIGIT expression on NK cells was not associated with the control of HIV-1 replication during the acute phase of HIV-1 infection.

### Reduced CD96^–^CD226^+^NK Cells and Expanded CD96^+^CD226^–^NK Cells had Opposite Effects on Viral Load, as Well as CD4 T-Cell Counts

**Figure 2:**
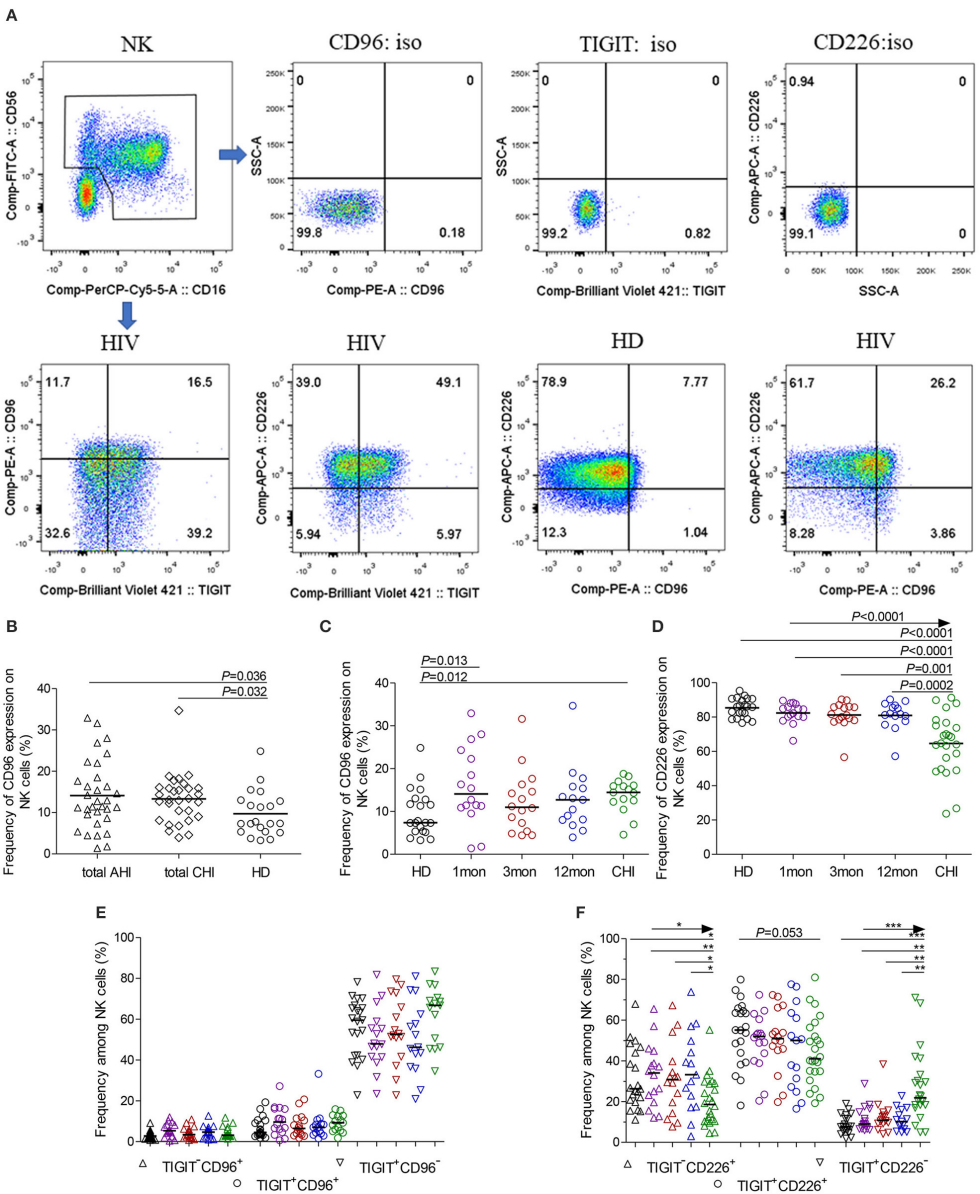
CD96, CD226 expression on NK cells at different stages of HIV-1 infection. **(A)** Flow cytometer charts of CD96 andCD226 expression on NK cells; **(B)** Comparison of CD96 expression on NK cells in both acute and chronic HIV-1 infection; **(C,D)** Change of CD96, CD226 expression on NK cells at different stages of infection; **(E,F)** Chang of TIGIT and CD96, TIGIT and CD226 coexpression on NK cells at different stages of infection; Arrow means the multiple groups comparison by the Kruskal-Wallis test; *P < 0.05; **P < 0.01; ***P < 0.001; total AHI: totally including the first and third month of acute HIV-1 infection; total CHI: totally including the twelfth month of infection and chronic HIV-1 infection over 2 years; HD: healthy donors; 1, 3, 12mon, CHI: the first, third, twelfth month of HIV-1 infection, and chronic HIV-1 infection over 2 years, respectively.

CD96 expression on NK cells, as shown in the flow cytometer charts of Figure 2A, was significantly upregulated in total acute and chronic HIV-1 infection (Figure 2B, *P* = 0.036, *P* = 0.032, respectively). In detail, CD96 expression on NK cells was transiently and significantly upregulated in the first month after HIV-1 infection (Figure 2C, *P* = 0.013) but not in the third and twelfth month of infection, and then the significant CD96 upregulation on NK cells was exhibited in chronic infection over 2 years (Figure 2C, *P* = 0.012). Besides, the number of TIGIT^+^CD96^+^NK cells was far fewer than TIGIT^+^CD96^−^NK cells in HIV-1-infected individuals and healthy donors, as shown in Figure 2E.

Different from TIGIT expression on NK cells, CD226 expression on NK cells significantly decreased in chronic infection over 2 years (Figure 2D, *P* < 0.0001) as compared to healthy individuals. CD226^+^NK cells in chronic infection over 2 years were fewer than that in the first, third, twelfth month after HIV-1 infection (Figure 2D, all *P* < 0.05). TIGIT and CD226 coexpression on NK cells was downregulated until in chronic HIV-1 infection over 2 years, though it is insignificant (Figure 2F, *P* = 0.053). Meanwhile, TIGIT^−^CD226^+^NK cells significantly diminished (all *P* < 0.05) but TIGIT^+^CD226^−^NK cells increased (all *P* < 0.01) in chronic infection over two years as compared with HIV-1-infected individuals in the first, third, twelfth month after HIV-1 infection and healthy individuals, as shown in Figure 2F.

**Figure 3:**
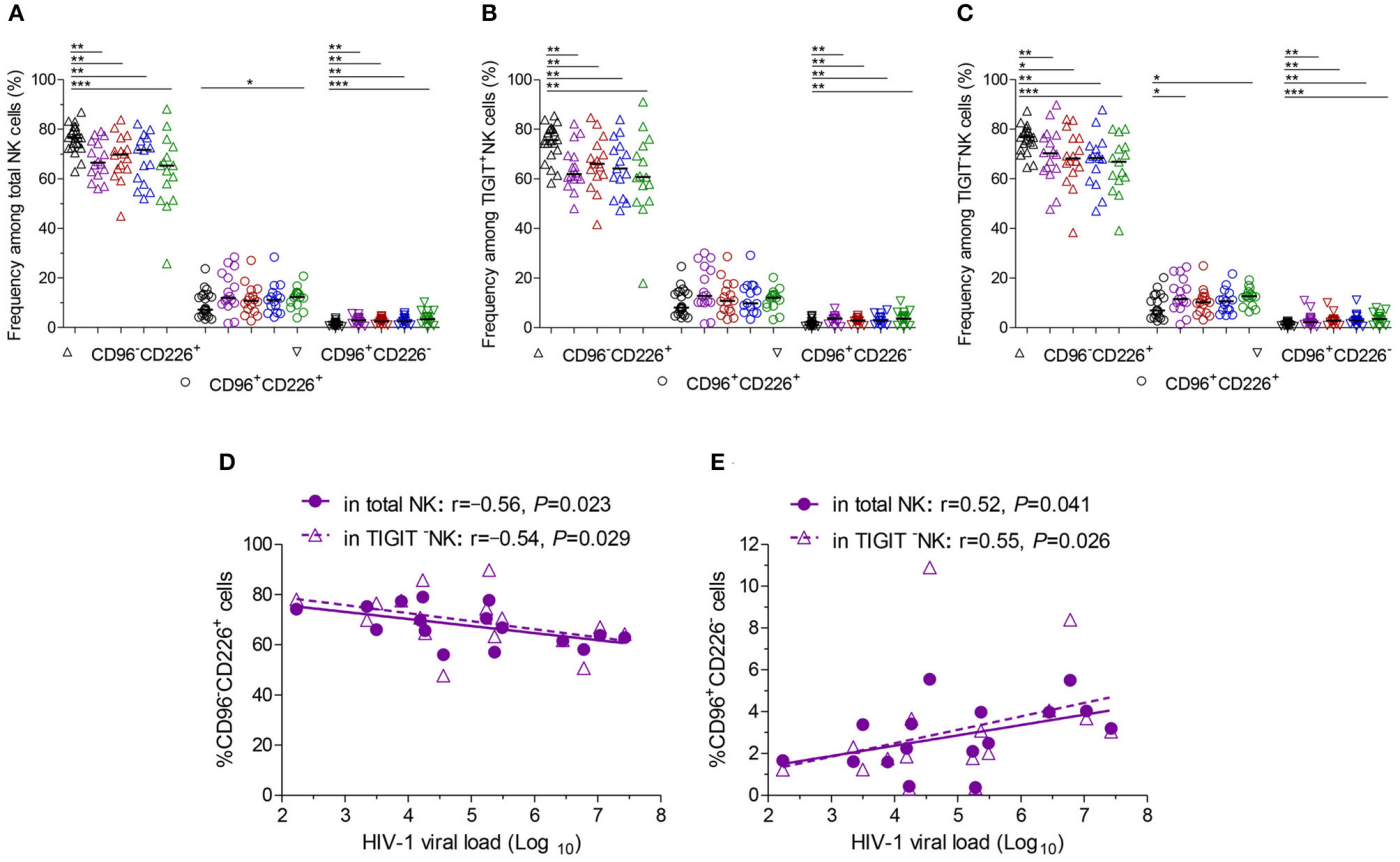
CD96 and CD226 coexpression on NK cells at different stages of HIV-1 infection. **(A)** CD96 and CD226 coexpression on total NK cells; **(B)** CD96 and CD226 coexpression on TIGIT^+^NK cells; **(C)** CD96 and CD226 coexpression on TIGIT^−^NK cells; **(D)** The correlation between the levels of HIV-1 viral load in the first month after HIV-1 infection and the amounts CD96^−^CD226^+^ cells among total NK cells, TIGIT^−^NK cells; **(E)** The correlation between the levels of HIV-1 viral load in the first month after HIV-1 infection and the amounts CD96^+^CD226^−^ cells among total NK cells, TIGIT^−^NK cells; Healthy donors, the HIV-1-infected individuals in the first, third, twelfth month of HIV-1 infection and chronic HIV-1 infection over 2 years were presented in black, purple, red, blue and green, respectively. *P < 0.05; **P < 0.01; ***P < 0.001.

Additionally, at different stages of infection, CD96^−^CD226^+^ cells among NK cells, TIGIT^+^NK and TIGIT^−^NK cells significantly diminished, while CD96^+^CD226^−^ cells expanded (Figures 3A–C, all *P* < 0.05). CD96^+^CD226^+^ cells among NK cells especially TIGIT^+^NK cells did not significantly expand at different stages of infection (Figure 3B), while these cells among TIGIT^−^NK cells increased in the first month of infection and in chronic infection over 2 years (Figure 3C, all *P* < 0.05). Strikingly, in the first month of infection, viral load was inversely associated with the number of CD96^−^CD226^+^NK cells among NK cells especially TIGIT^−^NK cells (Figure 3D, *r* = −0.56, *P* = 0.023; *r* = −0.54, *P* = 0.029, respectively), but positively associated with the amounts of CD96^+^CD226^−^ cells among NK cells especially TIGIT^−^NK cells (Figure 3E, *r* = 0.52, *P* = 0.041; *r* = 0.55, *P* = 0.026, respectively). Besides, in including the twelfth month and over 2 years of chronically infected HIV-1 individuals, CD4 T-cell counts were positively associated with the amounts of CD96^−^CD226^+^ cells in NK cells including TIGIT^+^NK and TIGIT^−^NK cells, but inversely with the amounts of CD96^+^CD226^−^ cells in NK cells including TIGIT^+^NK and TIGIT^−^NK cells (Table 1). These results suggest that reduced CD96^−^CD226^+^NK cells and augmented CD96^+^CD226^−^NK cells played opposite roles in suppressing HIV-1 replication after the onsetting of HIV-1 infection, and then had opposite effects on CD4 T-cell counts in persistent HIV-1 infection.

**Table 1:** Relationship between two variables.

	**1mon**		**3mon**		**12mon**		**CHI**		**Total AHI**		**Total CHI**		**HD**	
	***r***	***P***	***r***	***P***	***r***	***P***	***r***	***P***	***r***	***P***	**r**	***P***	**r**	***P***
CD96^−^CD226^+^NK vs. CD4	0.50	0.0486	^**_**^	^**_**^	0.50	*0.058*	^**_**^	^**_**^	^**_**^	^**_**^	0.52	0.005	NA	NA
CD96^−^CD226^+^/TIGIT^+^NK vs. CD4	0.45	*0.079*	^**_**^	^**_**^	^**_**^	^**_**^	^**_**^	^**_**^	^**_**^	^**_**^	0.46	0.017	NA	NA
CD96^−^CD226^+^/TIGIT^−^NK vs. CD4	^**_**^	^**_**^	^**_**^	^**_**^	0.47	*0.078*	^**_**^	^**_**^	^**_**^	^**_**^	0.36	*0.062*	NA	NA
CD96^+^CD226^−^NK vs. CD4	−0.41	*0.109*	^**_**^	^**_**^	−0.57	0.026	^**_**^	^**_**^	^**_**^	^**_**^	−0.57	0.002	NA	NA
CD96^+^CD226^−^/TIGIT^+^NK vs. CD4	−0.43	*0.092*	^**_**^	^**_**^	^**_**^	^**_**^	^**_**^	^**_**^	^**_**^	^**_**^	−0.52	0.006	NA	NA
CD96^+^CD226^−^/TIGIT^−^NK vs. CD4	^**_**^	^**_**^	^**_**^	^**_**^	−0.52	0.047	^**_**^	^**_**^	^**_**^	^**_**^	−0.44	0.021	NA	NA
CD155^+^CD4 vs. CD4	−0.76	0.0006	^**_**^	^**_**^	^**_**^	^**_**^	−0.56	0.004	−0.53	0.0017	−0.54	0.0004	NA	NA
NKG2A^−^NKG2C^+^NK vs. VL	0.54	0.025	0.44	*0.063*	-	-	-	-	0.44	0.007	-	-	NA	NA
NKG2A^+^NK vs. NKG2C^+^NK	-	-	-	-	−0.59	0.008	−0.58	0.015	-	-	−0.59	0.0001	-	-
NKG2A^−^NKG2C^+^NK vs. NKG2A^+^NKG2C^−^NK	-	-	-	-	−0.58	0.009	−0.68	0.003	-	-	−0.62	<0.0001	-	-
TIGIT^+^NK vs. NKG2C^+^NK	0.65	0.005	0.53	0.02	0.64	0.003	-	-	0.59	<0.0001	0.37	0.025	0.35	0.024
TIGIT^+^NK vs. NKG2A^−^NKG2C^+^NK	0.66	0.004	0.57	0.011	0.65	0.003	-	-	0.62	<0.0001	0.37	0.023	0.36	0.02
CD69^+^NK vs. VL	0.59	0.013	0.5	0.031	0.61	0.006	-	-	0.52	0.0009	-	-	NA	NA
CD69^+^/TIGIT^+^NK vs. VL	-	-	-	-	0.44	*0.062*	-	-	0.33	*0.052*	-	-	NA	NA
CD69^+^/TIGIT^−^NK vs. VL	0.65	0.005	0.49	0.035	0.52	0.023	-	-	0.53	0.001	-	-	NA	NA
CD69^+^NK vs. CD4	−0.71	0.001	-	-	-	-	-	-	−0.49	0.002	−0.4	0.016	NA	NA
CD69^+^/TIGIT^+^NK vs. CD4	−0.72	0.001	-	-	-	-	-	-	−0.49	0.002	−0.35	0.037	NA	NA
CD69^+^/TIGIT^−^NK vs. CD4	−0.66	0.004	-	-	-	-	-	-	−0.5	0.002	−0.41	0.012	NA	NA
CD69^+^NK vs. TIGIT^+^NK	-	-	0.48	0.038	-	-	-	-	0.43	0.009	0.35	0.039	0.44	0.006
CD38^+^HLA-DR^+^NK vs. VL	0.54	0.026	0.49	0.032	0.48	0.037	0.53	0.03	0.49	0.002	0.61	0.0001	NA	NA
CD38^+^HLA-DR^+^TIGIT^+^NK vs. VL	0.43	*0.086*	0.46	0.045	0.41	*0.085*	0.52	0.032	0.39	0.018	0.56	0.0006	NA	NA
CD38^+^HLA-DR^+^TIGIT^−^NK vs. VL	0.52	0.031	0.48	0.037	0.58	0.009	0.57	0.016	0.48	0.003	0.62	<0.0001	NA	NA
CD38^+^HLA-DR^+^NK vs. CD4	−0.45	*0.068*	−0.51	0.025	−0.61	0.005	-	-	−0.43	0.008	−0.43	0.011	NA	NA
CD38^+^HLA-DR^+^TIGIT^+^NK vs. CD4	−0.6	0.011	−0.46	0.046	−0.54	0.018	-	-	−0.48	0.003	−0.34	0.048	NA	NA
CD38^+^HLA-DR^+^TIGIT^−^NK vs. CD4	−0.5	0.043	−0.49	0.033	−0.56	0.012	-	-	−0.45	0.006	−0.41	0.017	NA	NA
CD38^+^HLA-DR^+^NK vs. TIGIT^+^NK	-	-	0.64	0.003	-	-	-	-	0.5	0.002	0.34	*0.052*	-	-
Ki67^+^NK vs. VL	0.5	0.041	0.48	0.04	0.66	0.002	-	-	0.46	0.005	0.48	0.003	NA	NA
Ki67^+^NK vs. CD4	−0.65	0.005	-	-	-	-	-	-	−0.51	0.002	-	-	NA	NA
Ki67^+^TIGIT^+^NK vs. CD4	−0.63	0.007	-	-	-	-	-	-	−0.49	0.003	-	-	NA	NA
Ki67^+^TIGIT^−^NK vs. CD4	−0.68	0.003	-	-	-	-	-	-	−0.54	0.001	-	-	NA	NA

*1mon, 3mon, 12mon, CHI, total AHI, total CHI and HD: represent the first, third, twelfth month of HIV-1 infection, chronic HIV-1 infection over 2 years, totally including the first and third month of acute HIV-1 infection, totally including the twelfth month of infection and chronic HIV-1 infection over 2 years, and HD for healthy donors, respectively; Spearman correlation test was used to analyze the relationship between 2 variables; r: correlation coefficient; P, P-value; VL, HIV-1 viral load (log_10_); CD4, CD4 T-cell count; ^**_**^, The correlation between two variables is insignificant and the figures including coefficient and P-value are not presented; The correlation trend toward statistically significant between two variables is shown in italic type; NA, not applicable*.

### CD155 Expression on CD4 T Cells Was Inversely Associated With CD4 T-Cell Counts in HIV-1 Infection

Although CD155 expression on CD4 T cells was very few as shown in Supplementary Figure 1A, it was significantly upregulated in the first, twelfth month of infection and in chronic infection over 2 years as compared with healthy individuals (Supplementary Figure 1B, all *P* < 0.05). CD155 expression on CD4 T cells was inversely with CD4 T-cell counts in acute HIV-1 infection (Supplementary Figure 1C, *r* = −0.53, *P* = 0.0017) including the first, third month of infection, and in chronic HIV-1 infection (Supplementary Figure 1D, *r* = −0.54, *P* = 0.0004) including the twelfth month, over 2 years of infection.

### NKG2A^−^NKG2C^+^ NK Cells Harbored more TIGIT-Expressing Cells than NKG2A^+^NKG2C^−^NK Cells

**Figure 4:**
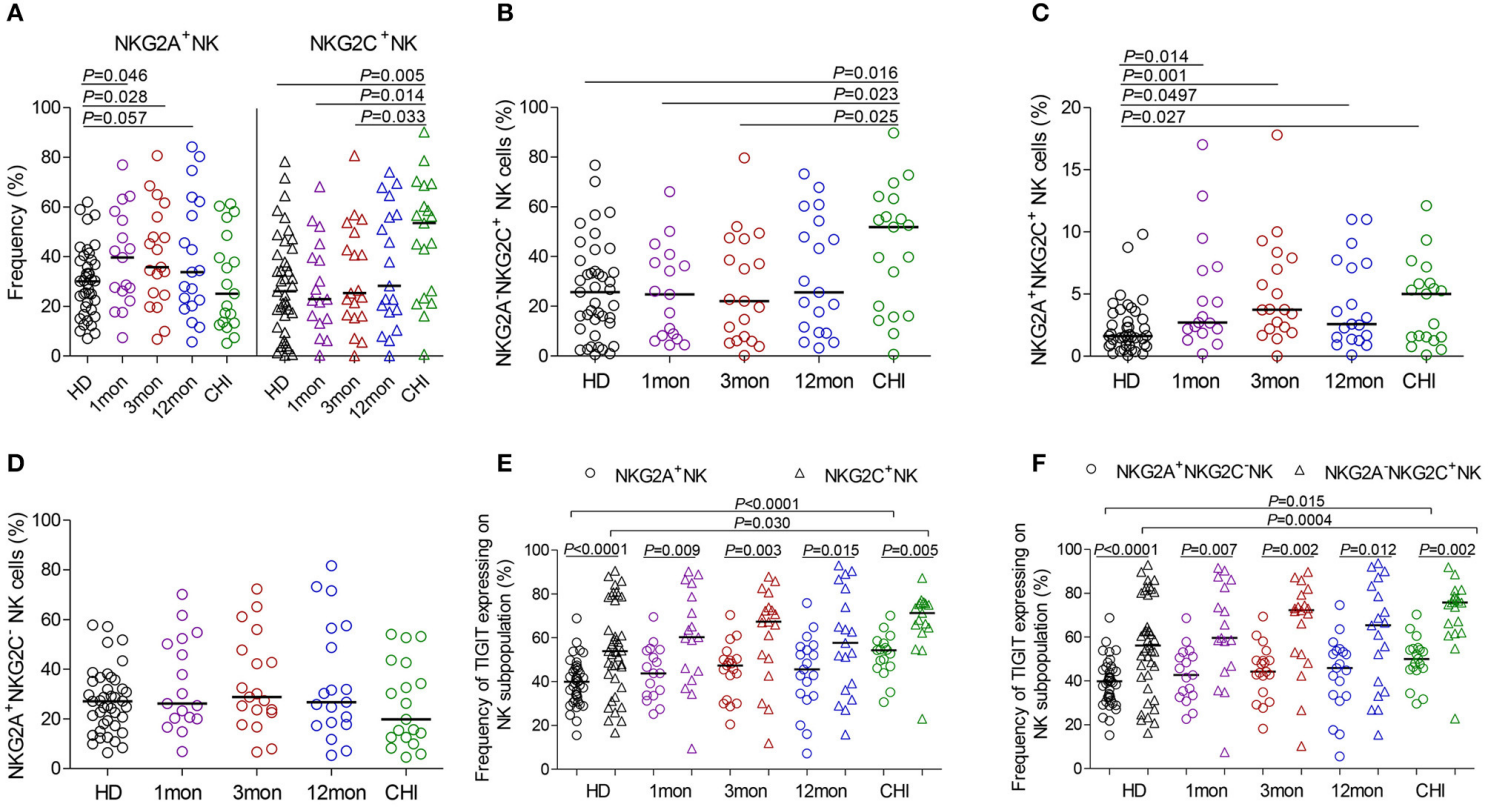
NKG2A, NKG2C and TIGIT expression on NK cell subsets at different stages of HIV-1 infection. **(A)** Frequency of NKG2A and NKG2C expression on NK cells; **(B–D)** Difference of NKG2A and NKG2C expression on NK cells; **(E)** Comparison of the expression of TIGIT on NKG2A^+^NK cells to NKG2C^+^NK cells; **(F)** Comparison of the expression of TIGIT on NKG2A^+^NKG2C^−^NK cells to NKG2A^−^NKG2C^+^NK cells; HD: healthy donors; 1, 3, 12mon, CHI the first, third, twelfth month of HIV-1 infection, and chronic HIV-1 infection over 2 years, respectively.

Compared with healthy individuals, NKG2A^+^NK cells expanded in the first (*P* = 0.046), third (*P* = 0.028) and twelfth month (*P* = 0.057) (Figure 4A) after HIV-1 infection but not in chronic HIV-1 infection over 2 years. Unexpectedly, the frequency of NKG2C^+^NK cells did not increase in the first and third months or after twelfth month after HIV-1 infection until reaching chronic HIV-1 infection over 2 years (Figure 4A, *P* = 0.005); the same situation was observed in NKG2A^−^NKG2C^+^NK subpopulation cells as shown in Figure 4B. The proportion of NKG2A^+^NKG2C^+^NK cells rather than NKG2A^+^NKG2C^−^NK cells increased in the first, third and twelfth month of HIV-1 infection and in chronic infection (Figures 4C,D; all *P* < 0.05) compared with healthy individuals.

Furthermore, there was an inverse relationship between the proportions of NKG2A^+^NKG2C^−^NK cells and NKG2A^−^NKG2C^+^NK cells in the twelfth month of infection (*r* = −0.58, *P* = 0.009; Table 1) and in chronic infection over 2 years (*r* = −0.68, *P* = 0.003; Table 1). These data showed that NKG2A^−^NKG2C^+^NK cells did not increase in acute HIV-1 infection and were, therefore, not contributing to restraining HIV-1 infection.

In HIV-1-negative individuals, the amounts of TIGIT^+^NK cells were positively associated with the frequency of NKG2A^−^NKG2C^+^NK cells (*r* = 0.36, *P* = 0.020; Table 1). Likewise, this positive correlation was observed in HIV-1-infected individuals in the first (*r* = 0.66, *P* = 0.004), third (*r* = 0.57, *P* = 0.011) and twelfth month (*r* = 0.65, *P* = 0.003) of HIV-1 infection but not in chronic HIV-1 infection over 2 years (Table 1). Compared with healthy donors, proportionally increased TIGIT expression on NK cell subpopulations, including NKG2A^+^NK cells, NKG2C^+^NK cells, NKG2A^+^NKG2C^−^NK cells and NKG2A^−^NKG2C^+^NK cells was only observed in chronic HIV-1 infection over 2 years (Figures 4E,F, all *P* < 0.05). Moreover, expression of TIGIT on NKG2A^−^NKG2C^+^NK cells was greater than that on NKG2A^+^NKG2C^−^NK cells in both HIV-1-infected individuals at different stages of infection as well as in healthy donors (Figures 4E,F, all *P* < 0.05). Since TIGIT is predominantly expressed on NKG2A^−^NKG2C^+^NK cells, it is understandable that NKG2A^−^NKG2C^+^NK cells in frequency are positively correlated with the levels of HIV-1 viral load in acute HIV-1 infection (Table 1), although they did not expand during acute HIV-1 infection but did so in chronic HIV-1 infection.

### Higher CD69 Expression on TIGIT^+^NK Cells and Association With CD4 T-Cell Loss

**Figure 5:**
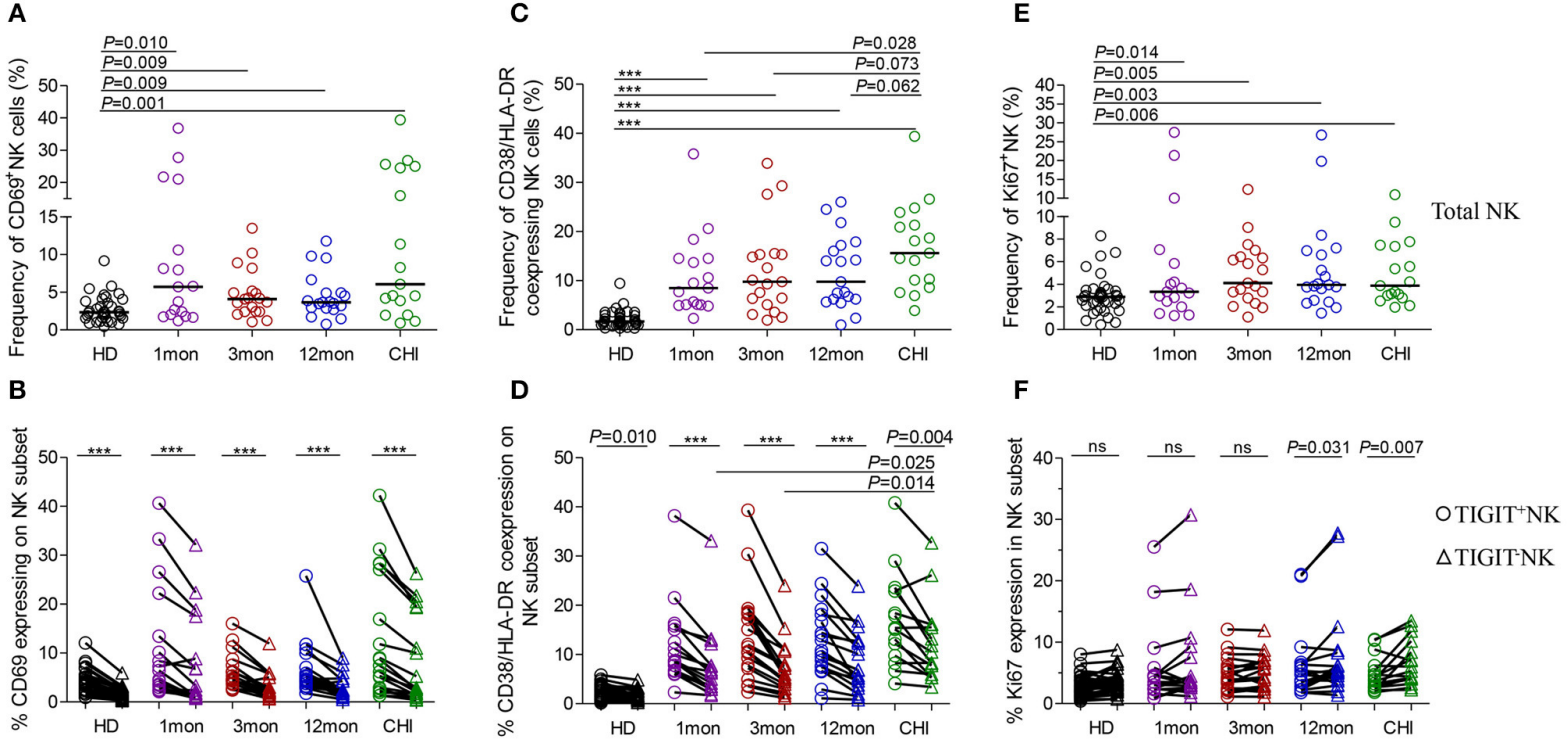
NK cell activation and proliferation at different stages of HIV-1 infection. **(A)** Capacity of NK cell early activation; **(B)** Difference of CD69 expression between TIGIT^+^NK cells and TIGIT^−^NK cells; **(C)** Frequency of CD38 and HLA-DR coexpressing NK cells; **(D)** Difference of CD38 and HLA-DR coexpression between TIGIT^+^NK cells and TIGIT^−^NK cells; **(E)** Capacity of NK cell proliferation; **(F)** Difference of TIGIT^+^NK and TIGIT^−^NK cell proliferation; 1, 3, 12mon, CHI: the first, third, twelfth month of HIV-1 infection, and chronic HIV-1 infection over 2 years, respectively; HD, healthy donors; Paired variables were statistically analyzed by Wilcoxon signed rank test; ***P < 0.001; ns: not significant.

CD69 is transiently expressed on activated leukocytes including T cells, B cells, NK cells and involved in early events of lymphocytes activation. Further, CD69 expression displays NK cell functional condition that associated with cytotoxic function (30). In our analysis, CD69 surface expression on NK cells was significantly higher in the first, third, and twelfth month, and in chronic infection over 2 years compared with HIV-1-negative donors (Figure 5A, all *P* < 0.01). Moreover, CD69^+^NK cells were positively associated with HIV-1 viral load in the first (*r* = 0.59, *P* = 0.013), third (*r* = 0.50, *P* = 0.031) and twelfth month (*r* = 0.61, *P* = 0.006) of infection but not in chronic HIV-1 infection over 2 years as shown in Table 1. An inverse association between the frequencies of CD69^+^NK cells and CD4 T-cell counts was shown in acute HIV-1 infection (*r* = −0.49, *P* = 0.002), especially in the first month of infection (*r* = −0.71, *P* = 0.001), as well as in including the twelfth month and more than 2 years of chronic HIV-1 infection (*r* = −0.40, *P* = 0.016).

Strikingly, the frequency of CD69 expression on TIGIT^+^NK cells at different stages of infection was significantly higher than the TIGIT^−^NK counterparts (Figure 5B; all *P* < 0.0001) and both subsets were higher than their respective HIV-1-negative donors (Figure 5B). In HIV-1 acute and chronic infection and even in healthy donors, the amounts of CD69-expressing NK cells were positively associated with the levels of TIGIT-expressing NK cells, as indicated in Table 1 (total AHI: *r* = 0.43, *P* = 0.009; total Chronic: *r* = 0.35, *P* = 0.039; HD: *r* = 0.44, *P* = 0.006). Also, the higher the levels of HIV-1 viral load, the more CD69-expressing TIGIT^+^NK cells and TIGIT^−^NK cells were detected at the acute stage of infection (TIGIT^+^NK: *r* = 0.33, *P* = 0.052; TIGIT^−^NK: *r* = −0.53, *P* = 0.001). In addition, the higher the frequencies of CD69-expressing TIGIT^+^NK and TIGIT^−^NK cells, the fewer CD4 T-cell counts were found in acute HIV-1 infection (TIGIT^+^NK: *r* = −0.49, *P* = 0.002; TIGIT^−^NK: *r* = −0.50, *P* = 0.002) and chronic HIV-1 infection (TIGIT^+^NK: *r* = −0.35, *P* = 0.037; TIGIT^−^NK: *r* = −0.41, *P* = 0.012). The above results demonstrate that following the onset of HIV-1 infection, early activation of NK cells, including TIGIT^+^NK cells and TIGIT^−^NK cells, was enhanced, which was affected by the levels of HIV-1 replication in acute infection. Then early activation of TIGIT^+^NK and TIGIT^−^NK cells may lead to the depletion of CD4 T cells in acute and chronic HIV-1 infection, especially in the first month of infection (TIGIT^+^NK: *r* = −0.72, *P* = 0.001; TIGIT^−^NK: *r* = −0.66, *P* = 0.004).

### Increased CD38/HLA-DR Coexpression on TIGIT^+^NK Cells and Association With a Decrease in CD4 T-Cell Counts

Lymphocytes can be activated within days of HIV-1 infection (31), where CD38 and HLA-DR molecules were upregulated on T lymphocytes. However, we also found increased CD38/HLA-DR coexpression on the surface of NK cells, including TIGIT^+^NK and TIGIT^−^NK cells, in the first, third and twelfth month of HIV-1 infection and in chronic infection over 2 years compared to healthy controls (Figure 5C, all *P* < 0.0001). Moreover, this phenomenon was found higher on chronic infection than acute infection (Figure 5C). Strikingly, the frequencies of CD38 and HLA-DR-coexpressing NK cells positively correlated with the levels of HIV-1 viral load but inversely related to CD4 T-cell counts in the first, third and twelfth month of HIV-1 infection, as depicted in Table 1.

Interestingly, CD38/HLA-DR coexpression on TIGIT^+^NK cell population was higher than the TIGIT^−^NK cell population in HIV-1-infected individuals who were at different phases of infection and in HIV-1-negative donors (Figure 5D, all *P* < 0.05). Persistent CD38 and HLA-DR coexpression on TIGIT^−^NK cells in over two years of chronic infection was significantly higher than in the first (*P* = 0.025) and third (*P* = 0.014) months of acute infection. Moreover, the frequency of CD38 and HLA-DR-coexpressing TIGIT^+^NK and TIGIT^−^NK cells, correlated positively with HIV-1 viral load, but had fewer CD4 T-cell counts in the first, third and twelfth month of infection (Table 1).

### TIGIT^+^NK Cells Showed Less Proliferative Capacities Than TIGIT^–^NK Cells in Chronic HIV-1 Infection

Proliferative NK cells were measured using Ki67 marker. NK cells including TIGIT^+^NK and TIGIT^−^NK cells showed significantly increased Ki67 expression in all HIV-1 infection status compared to HIV-1-negative donors (Figure 5E, all *P* < 0.05). As shown in Table 1, the amounts of proliferated total NK cells were positively associated with HIV-1 viral load in the first (*r* = 0.50, *P* = 0.041), third (*r* = 0.48, *P* = 0.040), and twelfth month (*r* = 0.66, *P* = 0.002) of infection and inversely correlated with CD4 T-cell counts but only in the first month of infection (*r* = −0.65, *P* = 0.005).

TIGIT^+^NK cells harbored proliferated cells comparable to the TIGIT^−^NK cells in HIV-1-negative donors and in HIV-1-infected individuals in the first and third months of infection but fewer expanded cells among TIGIT^−^NK cells in the twelfth month of infection (Figure 5F, *P* = 0.031) and in chronic infection over 2 years (Figure 5F, *P* = 0.007). As shown in Table 1, CD4 T-cell counts correlated inversely with the amounts of proliferated TIGIT^+^NK (*r* = −0.63, *P* = 0.007) and TIGIT^−^NK cells (*r* = −0.68, *P* = 0.003) only in the first month of infection. These results suggest that more proliferated TIGIT^+^NK and TIGIT^−^NK cells may be associated with increased CD4 T-cell loss, especially in the first month of infection.

### TNF-α, CD107a, and IFN-γ Released by TIGIT^+^NK Cells Were Lower Than TIGIT^–^NK Cells

**Figure 6:**
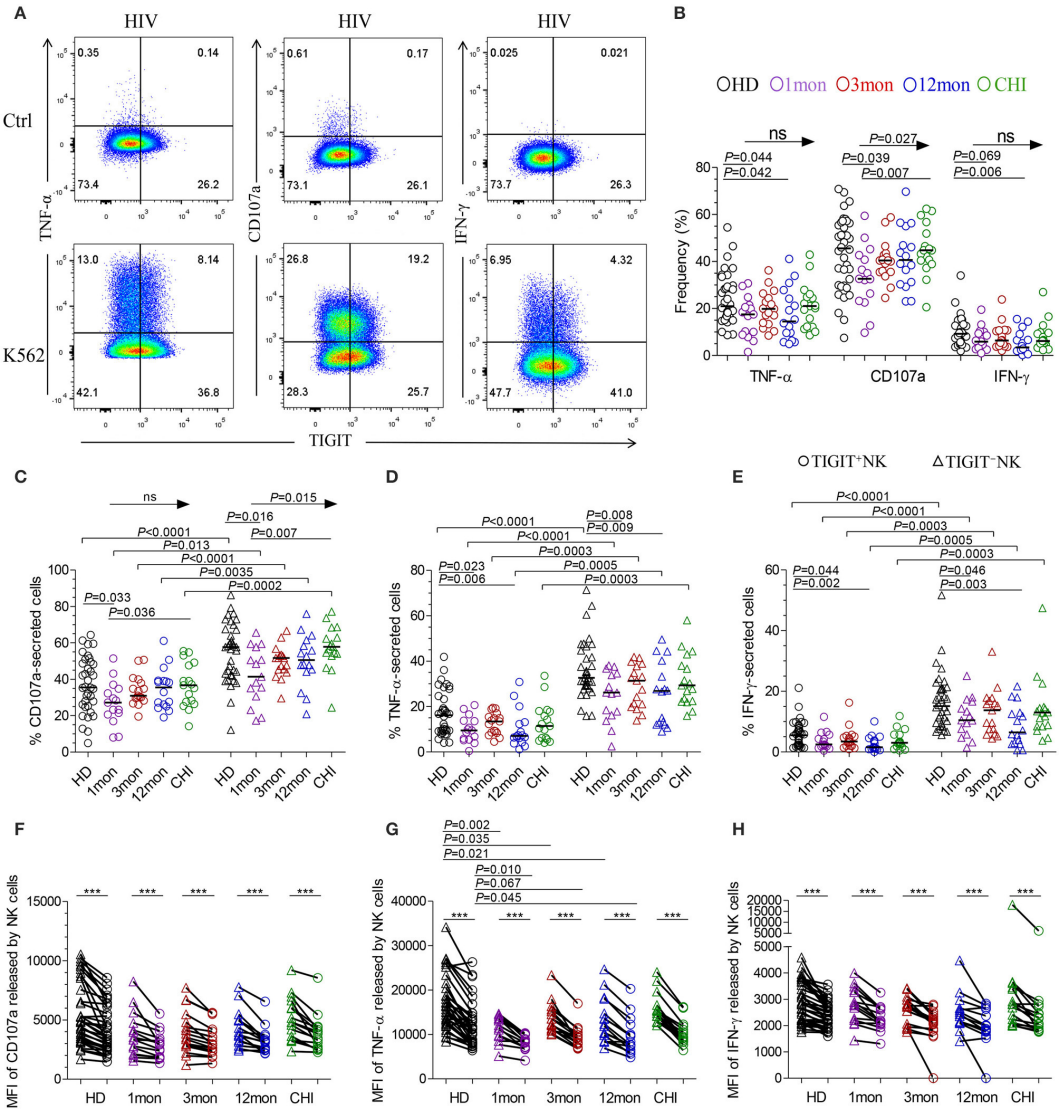
NK cell effector functions at different stages of HIV-1 infection. **(A)** Flow cytometer charts of analysis of NK cells producing CD107a, TNF-α and IFN-γ; **(B)** Comparison of the capacities of NK cells which secreted CD107a, TNF-α and IFN-γ; **(C–E)** Difference in the amounts of CD107a **(C)**, TNF-α **(D)**, IFN-γ **(E)** releasing TIGIT^+^NK and TIGIT^−^NK cells; **(F–H)** Median fluorescence intensity (MFI) of CD107a **(F)**, TNF-α **(G)**, IFN-γ **(H)** released by NK cells; Ctrl: NK cells were incubated with only RPMI1640; K562: NK cells were incubated with K562 target cells; 1, 3, 12mon, CHI: the first, third, twelfth month of HIV-1 infection, and chronic HIV-1 infection over 2 years, respectively; HD, healthy donors; Arrow means the multiple groups comparison by the Kruskal-Wallis test; Paired variables were statistically analyzed by Wilcoxon signed rank test; ***P < 0.001; ns: not significant.

The flow cytometer charts (Figure 6A) showed the degranulation substance CD107a, a sensitive marker of NK cell activity (32), produced by NK cells in response to K562 tumor cells significantly increased as HIV-1 infection prolonged to the stage of chronic infection (*P* = 0.027, Kruskal–Wallis test); TIGIT^−^NK cells showed this change (*P* = 0.015, Kruskal-Wallis test), but TIGIT^+^NK cells did not (Figures 6B,C). Overall NK cells, including TIGIT^−^NK and TIGIT^+^NK cells, secreted lower CD107a in the first month of acute HIV-1 infection than in chronic HIV-1 infection over 2 years and HIV-1-negative donors (Figures 6B,C, all *P* < 0.05). Cytokines such as TNF-α and IFN-γ secreted by NK cells, including TIGIT^−^NK cells and TIGIT^+^NK cells, decreased in the first, twelfth month of HIV-1 infection compared with HIV-1-negative donors (Figures 6B,D,E). These results showed that the degranulation of CD107a, TNF-α and IFN-γ secretion of NK cells, including TIGIT^+^ and TIGIT^−^ NK cells, was decreased in early acute infection, and it was the degranulation of NK cells significantly enhanced only during chronic infection.

When examining TIGIT^+^NK cells, they harbored fewer functional cells that released CD107a, TNF-α, and IFN-γ than TIGIT^−^NK cells in both HIV-1-positive individuals in all infection stages and in HIV-1-negative donors (Figures 6C–E, all *P* < 0.05). In addition, the median fluorescence intensity (MFI) of TIGIT^+^NK cells that expressed TNF-α, CD107a, and IFN-γ was significantly weaker than that of TIGIT^−^NK cells (Figures 6F–H, all *P* < 0.05) in both HIV-1-negative donors and HIV-1-infected individuals at different stages of infection. The MFI of TNF-α released by TIGIT^+^NK and TIGIT^−^NK cells in the first, third and twelfth month of infection was lower than that in HIV-1-negative donors (Figure 6G). Furthermore, in including the first and third months of acute HIV-1 infection, more NK cells expressed TIGIT, fewer NK cells released TNF-α, and there was a lower MFI of TNF-α expressed by NK cells, especially in the third month of infection (Supplementary Figures 2A,B). However, these inverse relationships were not significant in including the twelfth month and more than 2 years of chronic HIV-1 infection (Supplementary Figures 2C,D). These findings demonstrate that TIGIT^+^NK cells produce less CD107a, TNF-α, and IFN-γ than TIGIT^−^NK cells.

### Polyfunctionality of TIGIT^+^NK Cells Was Weaker Than TIGIT^–^NK Cells Among HIV-1 Infection

**Figure 7:**
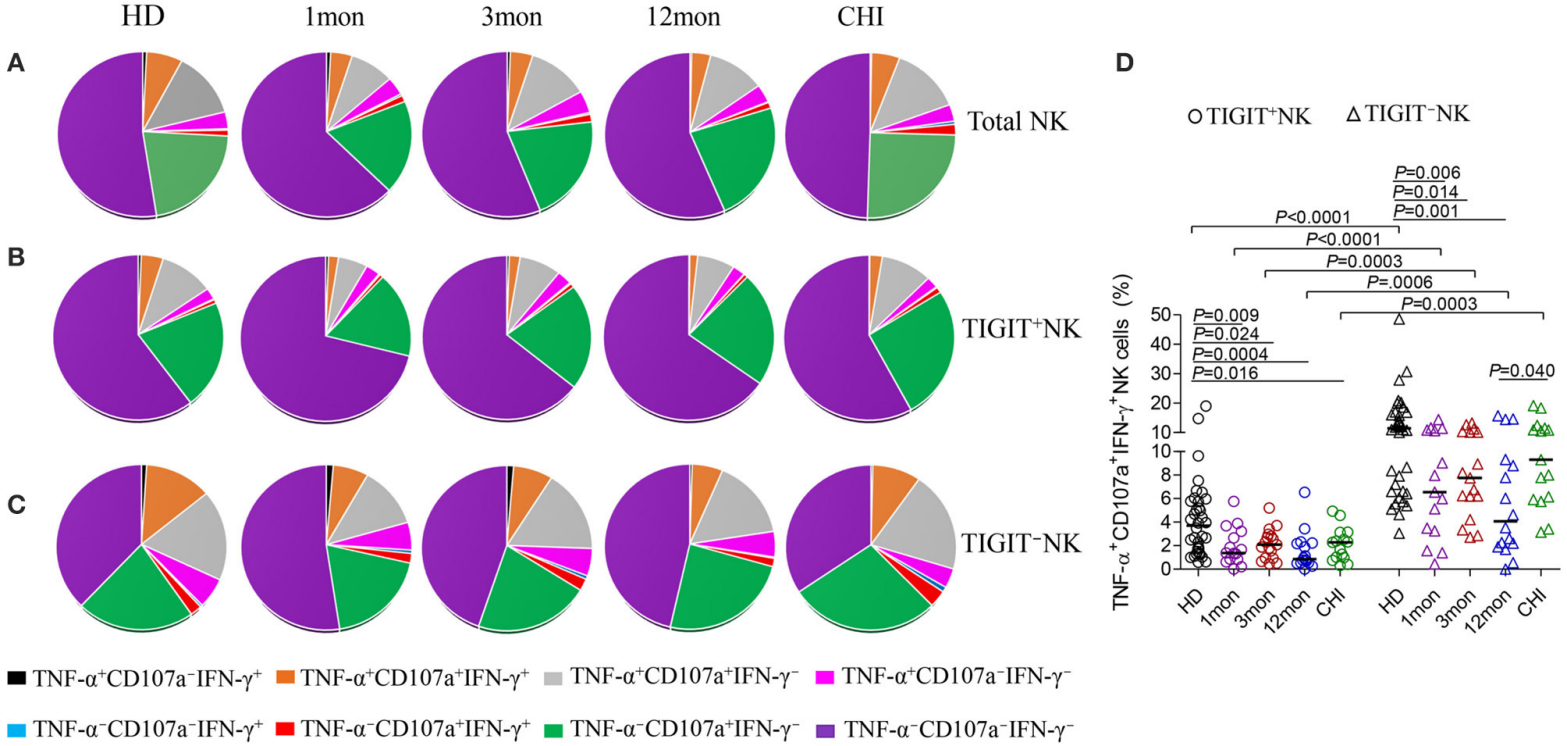
Comparison of the functionalities between TIGIT^+^NK and TIGIT^−^NK cells at different stages of HIV-1 infection. **(A)** Changes of the functionalities of total NK cells that secreted CD107a, TNF-α, IFN-γ; **(B)** Changes of the functionalities of TIGIT^+^NK cells during HIV-1 infection; **(C)** Changes of the functionalities of TIGIT^−^NK during HIV-1 infection; **(D)** Comparison of the levels of TNF-α^+^CD107a^+^IFN-γ^+^NK cells among TIGIT^+^NK cells and TIGIT^−^NK cells; 1, 3, 12mon, CHI: the first, third, twelfth month of HIV-1 infection, and chronic HIV-1 infection over 2 years, respectively; HD, healthy donors; The proportional average of functional NK cells is presented by pie chart area; Paired variables were statistically analyzed by Wilcoxon signed rank test.

From analysis for functional NK cells, the most common subsets were TNF-α^−^CD107a^+^IFN-γ^−^NK cells, followed by TNF-α^+^CD107a^+^IFN-γ^−^NK cells, TNF-α^+^CD107a^+^IFN-γ^+^NK cells and TNF-α^+^CD107a^−^IFN-γ^−^ NK cells (Figure 7A). This feature was also found for both TIGIT^+^NK and TIGIT^−^NK cells (Figures 7B,C). Interestingly, only the TNF-α^−^CD107a^+^IFN-γ^−^NK cells were comparable in frequency between TIGIT^+^NK and TIGIT^−^NK subpopulation cells in both HIV-1-infected individuals and healthy donors. In contrast, the other six types of functional cells, especially tri-functional cells among TIGIT^+^NK cells, were significantly reduced compared to TIGIT^−^NK cells in both HIV-1-infected individuals and healthy donors (Figure 7D, Wilcoxon signed rank test, all *P* < 0.01). Additionally, there were fewer functional TNF-α^+^CD107a^+^IFN-γ^+^NK cells among TIGIT^+^NK cells and TIGIT^−^NK cells in HIV-1-infected individuals in the first, third and twelfth month of infection than healthy donors (Figure 7D). Therefore, these data suggest that TIGIT expression on NK cells weakened the expression of CD107a, IFN-γ and TNF-α in response to K562 cells in both HIV-1-infected individuals and healthy donors. Additionally, the trifunctionality of TIGIT^+^NK and TIGIT^−^NK cells in secreting CD107a, TNF-α and IFN-γ might be decreased in both acute and chronic HIV-1 infection.

## Discussion

In this study, we analyzed and compared the characteristics of TIGIT^+^ and TIGIT^−^ NK cells at different stages of HIV-1 infection. Our data showed an increase in the frequency of TIGIT^+^NK cells were found in over 2 years of chronic HIV-1 infection but not in acute infection or even in early chronic infection. The amounts of TIGIT-expressing NK cells comparable to HIV-1-negative donors influenced HIV-1 viremia, in which more TIGIT-expressing NK cells resulted in a higher HIV-1 viral load in the first and third months of acute infection. These outcomes can be explained by Wang's report that in healthy individuals, a low-proportion TIGIT-expressing NK cells had higher degranulation activity, cytokine secretion capability, and cytotoxic potential than high-proportion TIGIT-expressing NK cells (17).

Contrary to the upregulated TIGIT expression on NK cells, CD96, which shares the ligand of CD155 with CD226 and TIGIT, was upregulated on NK cells in both acute and chronic HIV-1 infection. However, the expression of CD226 was not downregulated until in more than 2 years of chronic HIV-1 infection. In this study, CD96^−^CD226^+^ cells in total NK cells including TIGIT^+^NK and TIGIT^−^NK cells significantly diminished, while CD96^+^CD226^−^ cells expanded at all the acute and chronic phases of HIV-1 infection, which indicated that the expression of these two NK subset cells were oppositely affected. As opposed to CD226, CD96 and TIGIT are inhibitory receptors that counteract NK cell activation (23, 24). The opposite associations between HIV-1 viral load and the amounts of these two NK subset cells in the first month of infection indicate that diminished CD96^−^CD226^+^NK cells and expanded CD96^+^CD226^−^NK cells could play an antagonistic role. In which, CD96^−^CD226^+^NK cells were effective in suppressing HIV-1 replication but not CD96^+^CD226^−^NK cells. This opposite role was also presented in chronic HIV-1 infection because the associations between CD4 T-cell counts and the amounts of these two NK subset cells were antagonistic. In fact, CD96 and CD226 oppose each other in the regulation of NK cell functions. CD226 activates NK cell-mediated cytotoxicity, while CD96 inhibits NK cell function independently of CD226 (33). So, Further study will be needed to investigate the roles of these two NK subset cells during HIV-1 infection.

Although CD155, as the ligand of TIGIT, CD226 and CD96, is rarely expressed on CD4 T cells, it was upregulated in acute and chronic HIV-1 infection. Moreover, more CD4 T-cell-expressing CD155 was associated with lower CD4 T-cell counts, which implicates that CD155 expression on CD4 T cells might trigger NK cell activation and then reduce CD4 T cells. Indeed, CD155 and NKG2D ligands synergize as activating NK receptors to trigger NK cell lysis of the infected CD4 T cells (34).

NKG2A and NKG2C are rarely coexpressed on CD3^−^CD56^dim^ NK cells (35, 36), implying that NKG2A- and NKG2C-expressing NK cells may promote immune balance via their contradictory functions. In primary Epstein-Barr virus (EBV) infection, NKG2A^+^NK cells expand, particularly a population of early-differentiated NKG2A^+^KIR^−^CD56^dim^NK cells that do not contract and gradually acquire CD57 expression over time (37, 38). We also observed that NKG2A-expressing NK cells expanded after the onset of HIV-1 infection but that this gradually reduced with the prolongation of HIV-1 infection. In contrast, NKG2C-expressing NK cells, particularly the NKG2A^−^NKG2C^+^NK cell subpopulation, did not expand until in chronic HIV-1 infection over 2 years. NKG2A^+^NK cells expanded at the acute stage, but NKG2C^+^NK cells expanded at the chronic stage, suggesting that these two subsets may play different roles in different phases of HIV-1 infection. Of these, NKG2A^−^NKG2C^+^NK cells are not conducive to suppressing HIV-1 replication in acute HIV-1 infection, partly because the levels of TIGIT expressed on these adaptive NKG2A^−^NKG2C^+^NK cells were far higher than that on NKG2A^+^NKG2C^−^NK cells (Figure 4). Indeed, NKG2A^+^NK cells from HIV-1-uninfected individuals are implicated in better control of HIV-1 infection in *in vitro* models, as NKG2A^+^NK cells exhibit higher response and a polyfunctional profile of CD107a, IFN-γ, and CCL4 production in response to HLA-null cells and infected CD4^+^ T-cells compared to NKG2A^−^NK cells (39). Unfortunately, the human cytomegalovirus (CMV) status is not available from our study, but the CMV seroprevalence in Beijing is very low in HIV-1-infected patients in AIDS phase (40). Thus, we reasonably believe that the majority of the HIV-1-infected individuals were CMV seronegative, and that TIGIT upregulation in NKG2A^−^NKG2C^+^NK cells in chronic infection over 2 years was due to the direct effect of HIV infection rather than that of CMV infection.

Our data suggest a stronger CD69 expression on NK cells was associated with poor control of HIV-1 viremia and decrease of CD4 T-cell counts. In addition to its intrinsic value as an early activation marker, CD69 upregulation correlates with NK cytotoxicity and IFN-γ production (41, 42). CD69 surface expression on NK cells determines actual cytotoxic activity of peripheral blood NK cells. Therefore, the biological characteristics and functions of CD69 expression on TIGIT^+^NK cells in blood still need further investigation, especially in HIV-1 infection.

It is well established that the percentage of CD38^+^HLA-DR^+^CD8^+^ T cells correlates with plasma viral load and CD4 T-cell counts, the two parameters used in HIV-1 disease monitoring in the clinic (43–45). In this study, NK cell activation, as measured by CD38 and HLA-DR, correlated with markers of HIV-1 disease progression, similar to the findings reported by Kuri-Cervantes et al. (46). Contrary to CD8 T-cell activation, which increased promptly within days of HIV-1 invasion and then gradually decreased with the progression of HIV-1 infection (31), CD38 and HLA-DR coexpression by NK cells, including TIGIT^+^NK and TIGIT^−^NK cells, increased in the first month after HIV-1 infection and did not decrease even in chronic infection over 2 years. The activation capacity of CD8 T cells is affected by the HIV-1 viral load, and the levels of NK cell activation, including TIGIT^+^NK and TIGIT^−^NK cells, were also positively associated with HIV-1 viremia and inversely related to CD4 T-cell counts. Additionally, many more TIGIT^+^NK cells coexpressed CD38 and HLA-DR than TIGIT^−^NK cells in both HIV-1-infected individuals and healthy controls. These findings demonstrate that regardless of whether NK cells express TIGIT, increased activation of NK cells in HIV-1 infection was associated with CD4 T-cell depletion, despite stronger activation of TIGIT-expressing NK cells.

Contrary to NK cell activation aggravated by TIGIT expression in acute and chronic HIV-1 infection, TIGIT did not enhance NK cell proliferation. The reason is that comparable amounts of proliferated TIGIT^+^NK cells to TIGIT^−^NK cells were present in HIV-1-uninfected donors and HIV-1-infected individuals in the first and third months of acute infection, but with fewer proliferated TIGIT^+^NK cells than TIGIT^−^NK cells in the twelfth month of infection and in chronic infection over 2 years. Interestingly, especially in the first month of infection, the decrease in CD4 T-cell counts was related to the proliferation of NK cells, including both TIGIT^+^ and TIGIT^−^ subsets.

The effector capacity of NK cells in the context of HIV-1 infection is not restricted to cytotoxic elimination of target cells. NK cell activation may lead to the secretion of IFN-γ, TNF-α, and MIP-1β, influencing the antiviral response and limiting viral spread (47–49). The quantity, intensity and even multifunctionality of TIGIT^+^NK cells releasing CD107a, IFN-γ, and TNF-α in response to K562 cells were reduced compared to those of TIGIT^−^NK cells in both HIV-1-infected individuals and HIV-1-uninfected donors, suggesting that the potential effect of TIGIT-expressing NK cells on inhibiting HIV-1 infection might be weaker. Nonetheless, it will be worthwhile to study whether the responses of NK cells to HIV-1-infected CD4 T cells were dampened by TIGIT, and the effects of CD96, CD226 receptors on TIGIT^+^NK cells require further investigation. Moreover, Vendrame et al., reported that TIGIT expression is associated with increased NK responses to HIV-infected autologous CD4 T cells and K562 cell line in healthy donors (18), which differ from our findings that relatively weaker responses were triggered in TIGIT-expressing NK cells in both healthy donors and HIV-1-infected individuals. Therefore, large clinical samples would be needed to clarify the functions of TIGIT expression on NK cells during HIV-1 infection.

Taken together, our data provide an insight to the role of NK cells in the anti-HIV-1 immune response in relation to TIGIT expression. The phenotype and function of TIGIT^+^NK cells analyzed appear to be ineffective in controlling HIV-1 infection. Therefore, the findings serve as a basis for exploring the use of TIGIT^−^NK cells but avoiding TIGIT^+^NK cells in immunotherapy for HIV-1-infected patients for better clinical outcomes.

## Data Availability Statement

The original contributions presented in the study are included in the article/Supplementary Material, further inquiries can be directed to the corresponding author/s.

## Ethics Statement

All relevant experiments in this study were approved by the Beijing Youan Hospital Research Ethics Committee, and written informed consent was obtained from each participant in accordance with the Declaration of Helsinki. The patients/participants provided their written informed consent to participate in this study.

## Author Contributions

XZ and BS conceived the study and designed the experiments. XZ, XL, QZ, ZLiu, ZLi, LY, RW, YL, BT, and HX performed the experiments. HW, TZ, and BS contributed to reagents and materials and analyzed the data. XZ, AKLC, and BS wrote the article. BS supervised the whole study. All authors read and approved the final manuscript.

## Conflict of Interest

The authors declare that the research was conducted in the absence of any commercial or financial relationships that could be construed as a potential conflict of interest.

## Supplementary Material

Supplementary Figure 1 CD155 expression on CD4 T cells was negatively associated with CD4 T-cell counts. **(A)** Flow cytometer charts of CD155 expression on CD4 T cells; **(B)** Comparison of the change of CD155 expression on CD4 T cells at different stages of HIV-1 infection; **(C,D)** Inverse correlation of CD155 expression on CD4 T cells with CD4 T-cell counts in the first, third, twelfth month of infection and in chronic infection more than two years; Arrow means the multiple groups comparison by the Kruskal-Wallis test.

Supplementary Figure 2 Association of TIGIT expression with the levels of TNF-α secreted by NK cells. **(A)** Correlation between the amounts of TIGIT^+^NK cells and the levels of TNF-α secreting NK cells in the first, third month of infection; **(B)** Correlation between the amounts of TIGIT^+^NK cells and the MFI of TNF-α selected by NK cells in the first, third month of infection; **(C)** Correlation between the amounts of TIGIT^+^NK cells and the levels of TNF-α secreting NK cells in the twelfth month of infection and chronic HIV-1 infection over 2 years; **(D)** Correlation between the amounts of TIGIT^+^NK cells and the MFI of TNF-α selected by NK cells in the twelfth month of infection and chronic HIV-1 infection over 2 years; 1, 3, 12mon, CHI: the first, third, twelfth month of HIV-1 infection, and chronic HIV-1 infection over 2 years, respectively; Spearman correlation test was used to analyze the relationship between two variables.
