# Supplementary figures and images for: Analysis of the Characteristics of TIGIT-Expressing CD3−CD56+NK Cells in Controlling Different Stages of HIV-1 Infection
Source: Front Immunol. 2021 Feb 26;12:602492. doi: 10.3389/fimmu.2021.602492 (PMC7953050; doi:10.3389/fimmu.2021.602492)

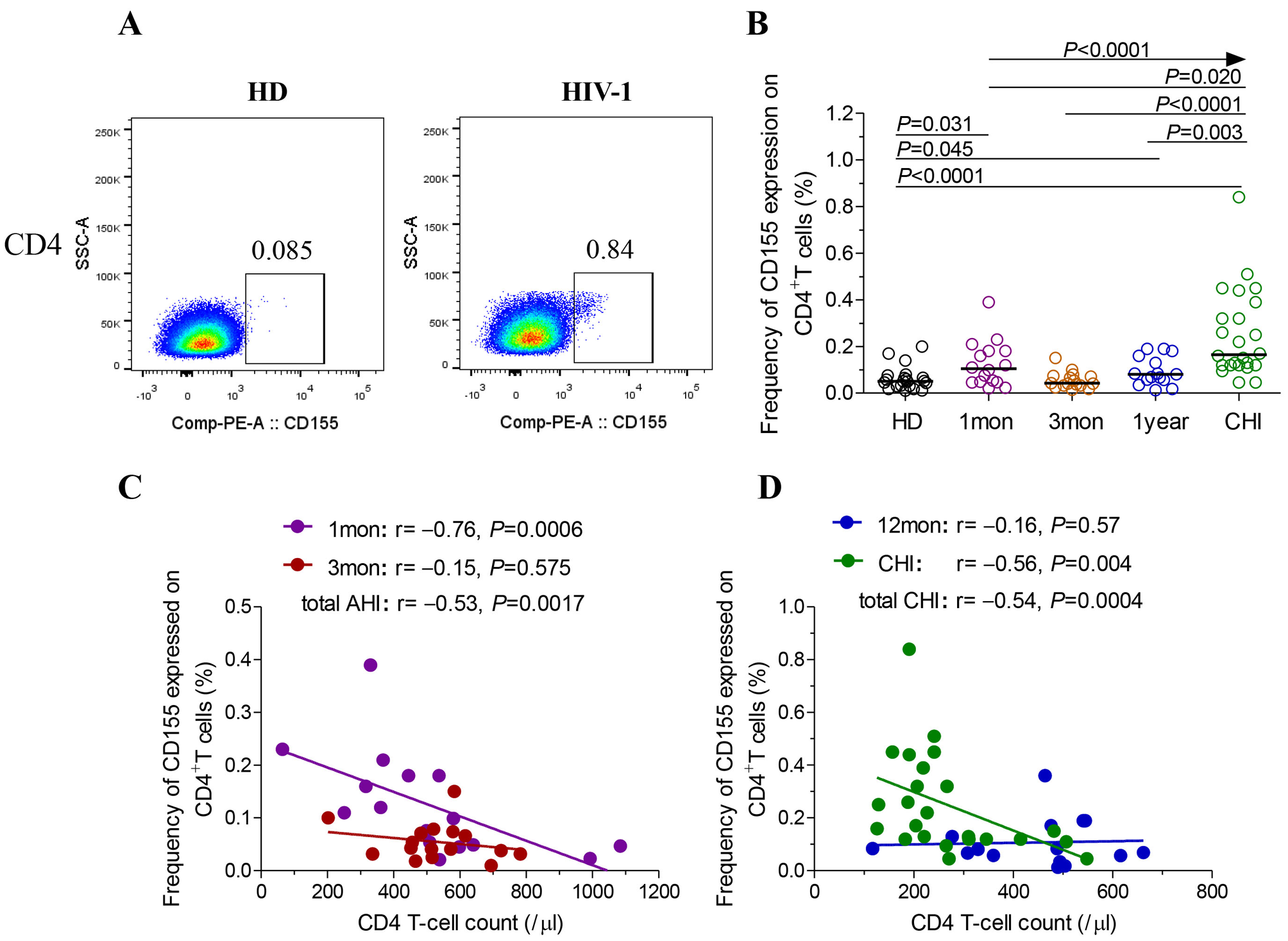

Supplement: Supplementary Figure 1 — CD155 expression on CD4 T cells was negatively associated with CD4 T-cell counts. (A) Flow cytometer charts of CD155 expression on CD4 T cells; (B) Comparison of the change of CD155 expression on CD4 T cells at different stages of HIV-1 infection; (C,D) Inverse correlation of CD155 expression on CD4 T cells with CD4 T-cell counts in the first, third, twelfth month of infection and in chronic infection more than two years; Arrow means the multiple groups comparison by the Kruskal-Wallis test. [file Image_1.tif]

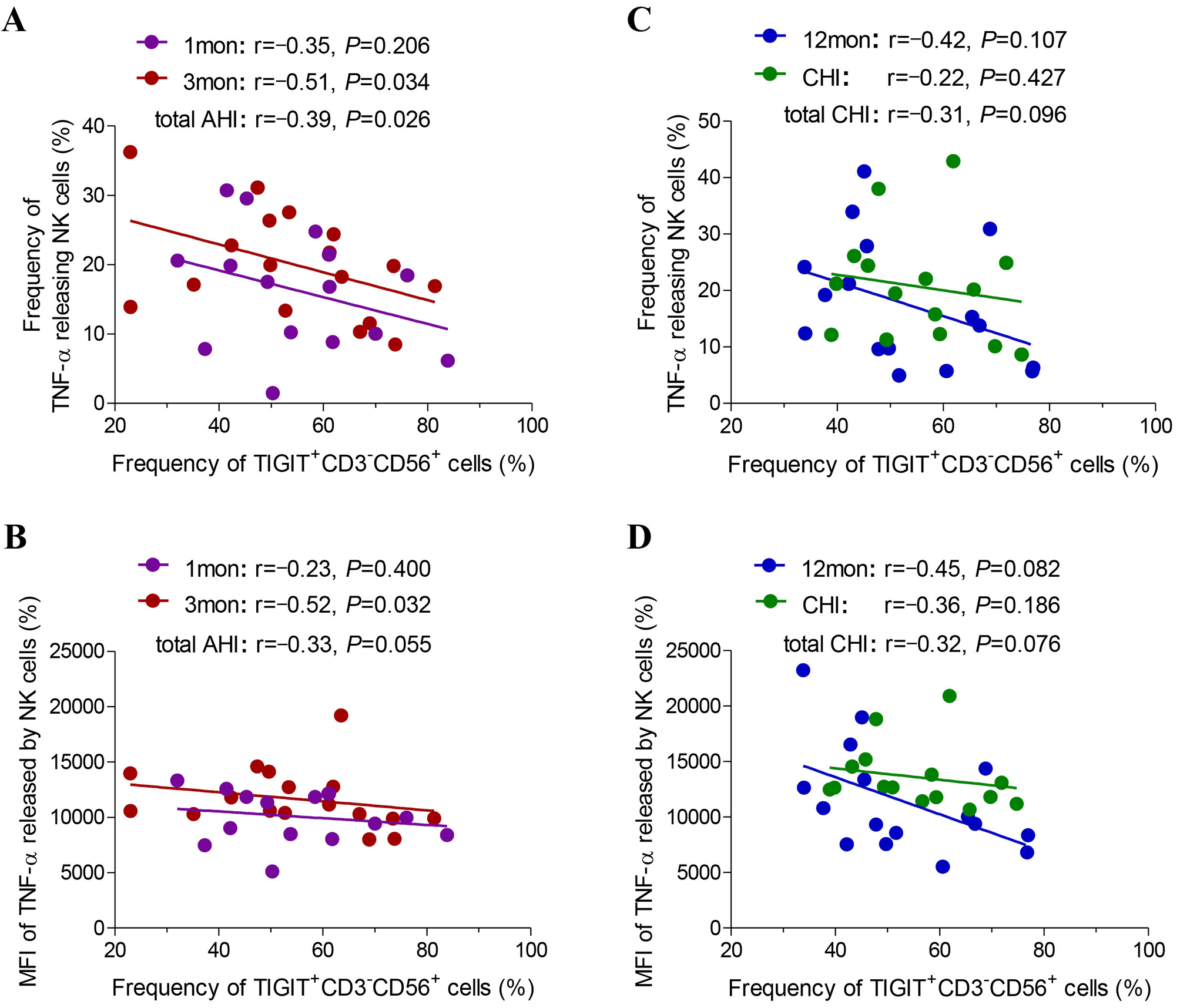

Supplement: Supplementary Figure 2 — Association of TIGIT expression with the levels of TNF-α secreted by NK cells. (A) Correlation between the amounts of TIGIT+NK cells and the levels of TNF-α secreting NK cells in the first, third month of infection; (B) Correlation between the amounts of TIGIT+NK cells and the MFI of TNF-α selected by NK cells in the first, third month of infection; (C) Correlation between the amounts of TIGIT+NK cells and the levels of TNF-α secreting NK cells in the twelfth month of infection and chronic HIV-1 infection over 2 years; (D) Correlation between the amounts of TIGIT+NK cells and the MFI of TNF-α selected by NK cells in the twelfth month of infection and chronic HIV-1 infection over 2 years; 1, 3, 12mon, CHI: the first, third, twelfth month of HIV-1 infection, and chronic HIV-1 infection over 2 years, respectively; Spearman correlation test was used to analyze the relationship between two variables. [file Image_2.tif]
